# Rate and correlates of self-stigma in adult patients with early psychosis

**DOI:** 10.3389/fpsyt.2023.1200568

**Published:** 2023-07-13

**Authors:** Ryan Sai Ting Chu, Chung Mun Ng, Sheung Chit Chu, Tsz Ting Lui, Fu Chun Lau, Sherry Kit Wa Chan, Edwin Ho Ming Lee, Christy Lai Ming Hui, Eric Yu Hai Chen, Simon Sai Yu Lui, Wing Chung Chang

**Affiliations:** ^1^Department of Psychiatry, School of Clinical Medicine, Li Ka Shing Faculty of Medicine, The University of Hong Kong, Hong Kong, Hong Kong SAR, China; ^2^State Key Laboratory of Brain and Cognitive Sciences, The University of Hong Kong, Hong Kong, Hong Kong SAR, China

**Keywords:** self-stigma, early psychosis, internalized stigma, duration of untreated psychosis, insight

## Abstract

**Introduction:**

Self-stigma impedes recovery process and is associated with poorer clinical and functional outcomes in people with psychotic disorders. However, there is limited research specifically examining self-stigma in the early stage of illness, and mixed findings were observed regarding factors associated with increased self-stigma. We aimed to investigate the rate and correlates of self-stigma in a cohort of adult patients with early psychosis using a comprehensive array of clinical, treatment and other illness-related variables.

**Methods:**

A total of 101 Chinese adult early psychosis patients aged 26–55 years who had received three-year psychiatric treatment for first psychotic episode in Hong Kong and completed self-stigma assessment were included for the current investigation. A broad range of assessments encompassing socio-demographics, premorbid adjustment, onset and illness profiles, symptom severity, psychosocial functioning, treatment characteristics and medication side-effects were conducted.

**Results:**

Twenty-eight (27.7%) patients had moderate-to-high levels of self-stigma. Univariate linear regression analyses showed that age at study entry, sex, educational level, age at psychosis onset, duration of untreated psychosis (DUP), insight level, global psychosocial functioning, and the use of second-generation antipsychotic were related to self-stigma levels. Final multivariable regression model revealed that female sex, younger age at entry, longer DUP and better insight were independently associated with higher levels of self-stigma.

**Conclusion:**

More than one-fourth of early psychosis patients experienced significant self-stigma, highlighting an unmet need for early detection and intervention of self-stigma in the initial years of illness. Further investigation is warranted to clarify trajectories and predictors of self-stigma in the early illness course.

## Introduction

Stigmatization refers to the process of labeling and stereotyping a group of people with undesirable characteristics, resulting in the negative consequence of separation, status loss and discrimination against these people (1). In general, stigma can be further categorized into several distinct but related subtypes, namely perceived public stigma, self-stigma and affiliate stigma. Public stigma denotes the endorsement of related stereotypes and prejudice as well as the manifestation of discrimination from the general public (2). Self-stigma is developed when individual with mental illness internalize the socially-endorsed negative stereotypes of the illness (2), while affiliate stigma refers to the caregivers’ internalized perceived public stigma toward patients (3). In particular, self-stigma has received increasing attention in psychiatric research, especially in people suffering from psychotic disorders. Literature has consistently indicated that a large proportion of patients with schizophrenia have experienced stigmatization and discrimination against their mental health condition during their lifetime (4). A recent meta-analysis reported that around one-third of schizophrenia patients exhibited high levels of self-stigma, with such undesirable situation being particularly common in southeastern Asian countries (5). Prior research suggested that greater self-stigma would impede treatment adherence and recovery process among patients with psychotic disorders (6–8). Accumulating data have further revealed that increased self-stigma may be associated with poor illness outcomes including greater symptom severity, elevated risk of psychiatric comorbidity, suicidal ideation and psychiatric hospitalization, reduced employment opportunity and lower functional status in patients with psychotic disorders (5).

Of note, the majority of prior studies examining self-stigma in psychotic disorders focused on patients with chronic illness (5, 9–11). Relatively few studies have systematically investigated stigmatization in the early course of psychotic disorder (12–19), and some of these studies in fact examined other forms of stigma rather than self-stigma in early psychosis patients, such as the disclosure form of stigma (16, 19) and perceived public stigma (15, 17, 18). We have previously observed significant association between perceived public stigma and self-stigma in patients with first-episode psychosis (FEP) (12). Our more recent study found that female sex, longer duration of untreated psychosis (DUP) and more severe positive symptoms at baseline were associated with greater self-stigma in early psychosis patients at 3-year follow-up (13). Some recent studies, albeit on chronic schizophrenia, have also suggested that patients with antipsychotic-induced motor side-effects may be more likely to experience increased self-stigma (20, 21). However, most earlier research, including our past studies, primarily focused on patients at younger age (12–16) (as early intervention services mainly treated young FEP patients). Given that there may be significant variations in illness presentations, treatment outcomes and service need between patients with typical age of onset (i.e., late adolescence or early adulthood) and those having their psychosis manifested at later years (22, 23), together with recent extension of some early intervention services to cover a wider adult age range (e.g., in United Kingdom and Hong Kong) (23, 24), more research is required to clarify self-stigma and its associated factors in early psychosis patients at higher-age groups.

To this end, the current study aimed to examine the prevalence and correlates of self-stigma in a cohort of Chinese adult patients with early psychosis. To ensure comprehensive evaluation of factors associated with self-stigma, a wide array of variables encompassing socio-demographics, premorbid adjustment, onset profile, symptomatology, treatment characteristics and side-effects were included in our analysis.

## Materials and methods

### Participants and study setting

A total of 130 Chinese patients aged 26–55 years presenting with first-episode non-affective psychosis (schizophrenia, schizoaffective disorder, schizophreniform disorder, brief psychotic disorder, delusional disorder or psychotic disorder not otherwise specified) to an extended EASY programme in Hong Kong were consecutively recruited from psychiatric outpatient units after they had completed the 3-year service (i.e., an early psychosis cohort). Briefly, EASY is a territory-wide publicly-funded early psychosis service, which has recently been expanded to provide 3-year care (originally as 2-year) to FEP patients aged 15–64 years (originally for 15–25 years) (25). The service provides an early assessment, and adopts phase-specific case-management approach in which each patient is assigned with a case manager who delivers protocol-based psychosocial interventions and assertively follows up patients for the first 3 years after their initial episode (25). Individuals with substance-induced psychosis, psychosis due to general medical condition, or intellectual disability were excluded. The study was approved by the local institutional review boards. Written informed consent were obtained from all subjects.

### Assessments

Diagnosis was ascertained by a senior research psychiatrist using the Chinese-bilingual Structured Clinical Interview for DSM-IV (CB-SCID) (26) and medical record review. Premorbid adjustment scale (PAS) (27) was used to measure premorbid functioning. Duration of untreated psychosis (DUP) and age of onset were determined using Interview for the Retrospective Assessment of the Onset of Schizophrenia (IRAOS) (28). Psychopathology was evaluated by positive and negative syndrome scale (PANSS) (29), brief negative symptom scale (BNSS) (30), and Calgary Depression Scale for Schizophrenia (CDSS) (31). Insight was assessed using Abbreviated Scale to Assess Unawareness of Mental Disorder (SUMD) (32), which measured awareness of mental disorder, illness consequences and medication effect. Psychosocial functioning was measured by social and occupational functioning assessment scale (SOFAS) (33). Antipsychotic-induced motor side-effects were examined by Simpson-Angus Scale (SAS) (34), Barnes akathisia rating scale (BARS) (35) and abnormal involuntary movement scale (AIMS) (36). Data on antipsychotic treatment and past history of psychiatric admission were also obtained. Data collection including clinical assessments were conducted by trained research assistants who received regular supervision and participated in biweekly rating consensus meetings throughout the study period to maintain quality assurance. Intraclass correlation coefficients for PANSS, BNSS, CDSS, and SUMD total scores ranged 0.82–0.91, indicating good interrater reliability.

Self-stigma of early psychosis patients was measured using the self-stigma scale-short form (SSS-S), which is a 9-item self-rated questionnaire covering affective, cognitive, and behavioral dimensions of self-stigma (37). Participants were asked to rate the extent to which they agreed with each item statement on a 4-point Likert scale from “1” strongly disagree to “4” strongly agree. A mean score was computed, with higher score indicating greater degree of self-stigma. Following the method of previous research (13, 38), a cutoff of mean score above the midpoint of 2.5 was adopted in defining the presence of moderate-to-high levels of self-stigma. This scale was validated and studied in Chinese patients with severe mental illness including chronic schizophrenia (37, 39) and early psychosis samples (12, 13), and was shown to have high convergent validity in relation to the key constructs that are closely associated with self-stigma (37).

### Statistical analysis

The primary analysis aimed to identify correlates of self-stigma. We adopted a two-step regression analysis approach with an aim to accommodate a large number of candidate variables, yet without compromising the statistical power of our study sample. First, a series of univariate linear regression analyses were performed to assess the associations of self-stigma with a comprehensive array of variables including socio-demographics, premorbid adjustment (PAS score), onset profile and illness characteristics (age at onset, DUP, diagnosis, past admission, family history of psychosis), symptom domains and psychosocial functioning (PANSS positive and disorganization scores, and total scores on BNSS, CDSS, SUMD, and SOFAS), and treatment characteristics and medication side-effects (antipsychotic type and dose, and scores on SAS, BARS, and AIMS). Then, those variables that showed *p*-value < 0.1 in the preceding univariable screening analyses (less stringent significance-level was adopted to minimize omitted-variable bias for subsequent multivariable analysis) were included in the final multivariable regression model to determine which factors were independently associated with self-stigma in early psychosis patients. DUP was log-transformed due to its skewed distribution. The level of statistical significance other than univariate analyses was set at *p* < 0.05.

## Results

### Characteristics of the sample

Table 1 summarizes the characteristics of the study sample. Of the initial cohort (*n* = 130), 101 participants completed the self-stigma assessment (i.e., SSS) and constituted the final sample for the study analysis. Among the 101 participants, 57.4% (*n* = 58) were female. The mean age of the sample was 40.5 years (S.D. = 7.8) and the median DUP was 151 days (mean = 417.3, S.D. = 624.4). Twenty-eight (27.7%) of participants were categorized as having moderate-to-high levels of self-stigma, and the mean self-stigma score was 2.3 (S.D. = 0.5).

**TABLE 1:** Demographic, clinical and treatment characteristics of the study sample.

Variables	Mean (SD)/*N* (%)
**Demographics**	
Age at study assessment	40.53 (7.79)
Female sex	58 (57.4)
Years of education	11.48 (3.50)
**Premorbid, onset and illness profiles**	
PAS overall score	0.32 (0.17)
Age at onset of psychosis	35.28 (8.17)
Duration of untreated psychosis, days^a^	151
**Psychiatric diagnosis^b^**	
Schizophrenia-spectrum disorder	64 (63.4)
Other non-affective psychoses	37 (36.6)
Past history of psychiatric admission	68 (67.3)
Family history of psychotic disorder	26 (25.7)
**Symptom and functional levels**	
PANSS positive symptom score^c^	8.82 (3.72)
PANSS disorganization score^c^	7.87 (1.45)
BNSS total score	12.30 (11.96)
CDSS total score	1.88 (2.94)
SUMD score	4.57 (1.83)
SOFAS score	60.88 (10.87)
**Treatment characteristics**	
Use of second-generation antipsychotic	84 (83.2)
Chlorpromazine equivalents, mg	419.63 (646.78)
SAS mean score	0.03 (0.14)
BARS global score	0.09 (0.47)
AIMS total score	0.27 (1.66)

^a^Duration of untreated psychosis (DUP) is presented in median.

^b^Schizophrenia-spectrum disorder included schizophrenia (*n* = 61) and schizoaffective disorder (*n* = 3). Other non-affective psychoses included delusional disorder (*n* = 9), brief psychotic disorder (*n* = 14) and psychosis not otherwise specified (*n* = 14).

^c^PANSS positive symptom and disorganization dimensions were derived on the basis of a previous factor-analytic study in early psychosis patients (65).

AIMS, abnormal involuntary movement scale; BARS, Barnes akathisia rating scale; BNSS, brief negative symptom scale; CDSS, Calgary Depression Scale for Schizophrenia; PANSS, positive and negative syndrome scale; PAS, premorbid adjustment scale; SAS, Simpson-Angus Scale; SOFAS, social and occupational functioning assessment scale; SUMD, Scale to Assess Unawareness of Mental Disorder.

### Factors associated with self-stigma

As shown in Table 2, univariate regression analyses showed that self-stigma score was associated with age at study assessment, sex, year of education, age of onset, DUP, SUMD score, SOFAS score and use of second-generation antipsychotic (SGA). A multivariable regression model revealed that younger age at study assessment, female gender, longer DUP and better insight (i.e., lower SUMD score) were independently associated with higher levels of self-stigma (Table 3).

**TABLE 2:** Univariate regression analyses of demographics, clinical and treatment variables for self-stigma (*N* = 101).

				95% CI	
Variables	B	t	*P*	Lower bound	Upper bound
**Demographics**					
Age at study assessment	-0.02	-4.14	<0.01	-0.03	-0.01
Female sex	0.23	2.59	0.01	0.05	0.41
Years of education	0.02	1.81	0.07	-0.01	0.05
**Premorbid, onset and illness profiles**					
PAS overall score	0.07	0.27	0.79	-0.48	0.63
Age at onset of psychosis	-0.02	-3.97	<0.01	-0.03	-0.01
Log DUP^a^	0.11	1.96	0.05	-0.01	0.23
Schizophrenia-spectrum disorder	0.11	0.95	0.35	-0.12	0.34
Past history of psychiatric admission	0.08	0.68	0.50	-0.13	0.26
Family history of psychotic disorder	-0.08	-0.76	0.45	-0.29	0.19
**Symptom and functional levels**					
PANSS positive symptom score	0.01	0.92	0.36	-0.01	0.04
PANSS disorganization score	-0.01	-0.20	0.84	-0.07	0.06
BNSS total score	0.01	1.16	0.25	-0.01	0.01
CDSS total score	0.03	1.60	0.11	-0.01	0.06
SUMD score	-0.05	-1.94	0.06	-0.10	0.01
SOFAS score	-0.01	-1.82	0.07	-0.02	0.01
**Treatment characteristics at study assessment**					
Use of second-generation antipsychotic	0.21	1.76	0.08	-0.03	0.45
Chlorpromazine equivalents, mg	0.01	1.38	0.17	0.001	0.001
SAS score	0.39	1.16	0.25	-0.28	1.07
BARS score	-0.09	-0.91	0.36	-0.28	0.11
AIMS score	-0.02	-0.53	0.60	-0.07	0.04

^a^DUP was log-transformed for analysis due to its skewed distribution.

AIMS, abnormal involuntary movement scale; BARS, Barnes akathisia rating scale; BNSS, brief negative symptom scale; CDSS, Calgary Depression Scale for Schizophrenia; DUP, duration of untreated psychosis; PANSS, positive and negative syndrome scale; PAS, premorbid adjustment scale; SAS, Simpson-Angus Scale; SOFAS, social and occupational functioning assessment scale; SUMD, Scale to Assess Unawareness of Mental Disorder.

**TABLE 3:** A final multivariable regression model for prediction of self-stigma^a,b^ (*N* = 101).

				95% CI	
Variables	B	t	*P*	Lower bound	Upper bound
Female sex	0.19	2.16	0.033	0.015	0.357
Age at study assessment	-0.07	-2.18	0.031	-0.139	-0.007
Log DUP	0.19	2.55	0.012	0.041	0.332
SUMD score	-0.05	-2.38	0.020	-0.099	-0.009

^a^Educational level, age at onset, SOFAS score and use of second-generation antipsychotic, which were included in the multivariable regression analysis, were excluded from the final model.

^b^Final model: adjusted *R*^2^ = 0.275, *F* = 6.416, *p* < 0.001.

DUP, duration of untreated psychosis; SOFAS, social and occupational functioning assessment scale; SUMD, Scale to Assess Unawareness of Mental Disorder.

## Discussion

The current study aimed to investigate the rate and correlates of self-stigma in a Chinese cohort of adult patients with early psychosis. Our results showed that approximately 28% of early psychosis patients exhibited moderate-to-high levels of self-stigma. Among various factors, female sex, younger age, longer DUP and better insight were found to be independently associated with greater self-stigma.

Our result on the prevalence of self-stigma in early psychosis patients largely accords with those observed in the literature on schizophrenia and other psychoses (ranged: 16.5 to 51%, with most studies reporting prevalence of self-stigma above 30%), with a recent meta-analysis estimated that 35.8% of schizophrenia patients displayed high self-stigma (5). Of note, it may be difficult to directly compare our data with previous findings due to significant cross-study methodological variation including the use of different assessment instruments for self-stigma and the heterogeneous characteristics of the patient samples (e.g., being evaluated in different stages of the illness) as earlier research primarily examined patients with chronic schizophrenia. Cross-cultural differences may also contribute to discrepant findings on self-stigma. Recent meta-analyses showed higher frequency of high self-stigma and greater stereotype endorsement in schizophrenia patients from Asian countries compared to the others (5, 40). Literature suggested that perceived stigma and self-stigma in people with mental illness may be greater in regions with relatively group-oriented cultures (e.g., Asian and Chinese populations) than those regions with more individual-oriented cultures (e.g., countries in Europe and North America) (41). However, as Hong Kong is a metropolitan city with comparatively more westernized culture, the effect of Chinese cultural factors on stigmatization in mental illness would likely be less pronounced than those regions associated with more traditional, collectivistic Chinese culture. Nevertheless, our results reveal that a significant proportion of patients experienced self-stigma in the early phase of psychotic disorder.

Many previous studies observed lack of sex difference in self-stigma among patients with severe mental illness including schizophrenia (5, 40). Our finding that female patients had greater self-stigma than the male counterparts is, however, in line with some earlier studies on chronic schizophrenia (42, 43), as well as young early psychosis sample in Hong Kong (13). Mixed findings were also noted in the association between age and self-stigma in patients with psychotic disorders. Many past reports revealed no significant association between age and self-stigma (5, 40). Nonetheless, our finding that younger patients had greater self-stigma than older patients concurs with some prior studies which demonstrated that self-stigma level increased with decreasing age of schizophrenia patients (44–46). It is plausible that patients are less exposed to stigmatization and its associated distress with increasing age because they might be more accustomed to these negative societal attitudes (45). Other investigators postulated that older patients may be able to cope with psychiatric symptoms more efficiently than younger patients (47), and such enhanced coping may help buffer the adverse effect of stigmatization (45, 48).

We found that patients with longer DUP had greater self-stigma. This is consistent with our previous study in young patients with early psychosis and a number of past reports on FEP samples (16, 17). In fact, prior research has suggested that perceived public stigma and self-stigmatization may increase the likelihood of delayed help-seeking and decreased use of psychiatric service (49), including first-episode patients (50). Alternatively, patients with prolonged DUP may experience a longer duration of exposure to perceived stigma, which in turn leads to increased susceptibility to internalization of stigma, relative to those with shorter DUP (13, 51). Hence, reduction of treatment delay by early detection of and timely intervention to initial psychosis would be critical for lowering the risk of self-stigmatization in early psychosis patients. In agreement with many previous studies examining correlates of self-stigma in psychotic disorders (5), our results demonstrated that higher level of insight was associated with greater self-stigma. Notably, a growing body of research has investigated the inter-relationship between self-stigma, insight and depression in patients with psychotic disorders, and has revealed that self-stigma mediates the association between insight and depression (52). Accumulating data further suggested that self-stigma may moderate the relationships between insight and other psychosocial variables. For instance, associations of good insight with increased demoralization, lower self-esteem, poorer life satisfaction and higher levels of hopelessness were stronger as self-stigma levels increased in schizophrenia patients (38, 53, 54). Given that insight to illness is an important therapeutic target in early intervention for psychosis, careful evaluation of the potential negative impact of improved insight on stigma internalization should be regularly conducted to minimize the risk of exacerbation of self-stigma levels among those patients with better insight. Conversely, contrary to some (4), though not all, past studies (12, 55–59), we did not find any significant association of self-stigma with positive, negative or depressive symptoms. One possible explanation is that our patient cohort were clinically stabilized with antipsychotic treatment, with relatively low symptom severity. The limited variance in symptom ratings may therefore obscure the potentially significant association between self-stigma and these symptom domains.

The current study has several important merits. First, this is among the few studies to examine self-stigma in the early course of psychotic disorders, with specific focus on adult patients with wider age range. Second, a comprehensive array of variables, including premorbid adjustment, onset profile, various symptom domains and treatment characteristics, were incorporated for analysis of correlates for self-stigma. However, several methodological limitations should be considered in interpreting the study results. First, the cross-sectional design precludes us from establishing causality between self-stigma and its associated correlates. Moreover, as this study recruited and assessed patients shortly after the end of the EASY programme, we were not able to evaluate the trajectory of patients’ self-stigma levels over time since service entry and the potential effect of early intervention on reducing self-stigma. Second, other variables that may be related to self-stigma, including cognitive functioning, meta-cognitive capacity, social perception, theory-of-mind abilities, illness appraisal and coping strategies, were not examined in this study. Third, SSS-S is a brief, albeit well-validated, scale for assessing self-stigma in patients with schizophrenia and related psychoses. Adoption of more comprehensive assessment scales such as internalized stigma of mental illness scale (ISMI) (60) and perceived devaluation and discrimination (PDD) (61) would provide a more refined evaluation of self-stigma and its related constructs in early psychosis. Fourth, this study only included adult patients aged 26–55 years, while the majority of early psychosis studies focused mainly on adolescent and young adult patients. This may therefore compromise the comparability of our study findings with the literature of early psychosis research.

In conclusion, our results show that more than one-fourth of Chinese adult early psychosis patients exhibited moderate-to-high levels of self-stigma. Female sex, younger age, longer DUP and better insight were independently associated with greater self-stigma. Owing to the fact that self-stigma is prevalent in the initial course of illness and may impede the recovery process, comprehensive assessment of self-stigma and regular monitoring of its adverse impact on clinical outcome and psychosocial functioning should be performed on a regular basis to facilitate early detection of and prompt interventions to patients at risk of developing high self-stigma. In fact, recent meta-analytic reviews have indicted efficacy of various psychological interventions in lowering self-stigma in patients with psychotic disorders (62, 63), and group therapies (such as assertive training and psychoeducation programs) may be particularly useful for patients with significant though relatively less severe self-stigma (62). A conceptual framework has also recently been proposed to adequately address stigma for early psychosis patients by delineating how distinct forms of stigma (including self-stigma and perceived public stigma) are linked to different treatment stages of early intervention service (64). This underscores an important unmet need to minimize self-stigma in early psychosis. Further research is warranted to unravel the longitudinal relationship between self-stigma and various illness and clinical variables so as to identify crucial treatment targets to alleviate self-stigma in the early phase of psychotic disorders.

## Data availability statement

The raw data supporting the conclusions of this article will be made available by the authors, without undue reservation.

## Ethics statement

The studies involving human participants were reviewed and approved by the Institutional Review Board of the University of Hong Kong/Hospital Authority Hong Kong West Cluster (HKU/HA HKW IRB). The patients/participants provided their written informed consent to participate in this study.

## Author contributions

WC designed and conceptualized the study. RC, CN, and SKWC conducted statistical analysis and wrote the first draft of the manuscript. WC, RC, and CN interpreted the study data. WC and RC revised and finalized the manuscript. All authors provided critical feedback to the manuscript and approved the final manuscript.
